# Prevalence, antimicrobial susceptibility, and genotyping of *Streptococcus agalactiae* in Tilapia fish (*Oreochromis niloticus*) in Egypt

**DOI:** 10.5455/javar.2022.i573

**Published:** 2022-03-10

**Authors:** Asmaa Alazab, Asmaa Sadat, Gamal Younis

**Affiliations:** Department of Bacteriology, Mycology and Immunology, Faculty of Veterinary Medicine, Mansoura University, 35516, Egypt

**Keywords:** *S. agalactiae*, Multi-drug resistance, Tilapia, Virulence genes, multiplex-PCR

## Abstract

**Objectives:**

Streptococcus agalactiae is a zoonotic human and animal pathogen that causes global economic losses in aquaculture and fatal outcomes in Tilapia. This study aimed to identify S. agalactiae isolated from different fish sources intended for human consumption phenotypically and genotypically and to characterize the virulence-associated genes *fbsA* (fibrinogen-binding protein FbsA), cfb (CAMP factor), and pbp1A/ponA (penicillin-binding protein 1A).

**Materials and Methods:**

Three hundred Nile Tilapia fish (*Oreochromis niloticus*) were collected from different farms and retail shops in Dakahlia and Damietta, Egypt, during the summer of 2020. The samples were examined using routine phenotypic methods, then characterized using polymerase chain reaction (PCR) targeting *S. agalactiae*-specific *dltS* gene. All S. agalactiae isolates were examined for the susceptibility to ten antimicrobial agents by the disc diffusion method. The virulence-associated genes (fbsA, cfb, and pbp1A/ponA) were characterized using multiplex-PCR.

**Results:**

Streptococcus agalactiae was detected in 7% (*n* = 21/300) samples. The isolates showed high resistance against ampicillin and erythromycin (20/21; 95%) for each. The most predominant antibiotypes through isolates were P, CN, SXT, CRO, TE, CTX, E, AMP, at 10.5% for each antibiotype. A total of 19 (90.5%) of S. agalactiae isolates showed multi-drug resistance (MDR), and those were recovered from market Tilapia fish. The virulence-associated genes (fbsA, cfb, and pbp1A/ponA) were identified in the S. agalactiae as 100%, 76%, and 52%, respectively.

**Conclusions:**

The MDR S. agalactiae detected in raw Tilapia fish pose a significant health hazard to consumers due to their zoonotic characteristics.

## Introduction

Streptococcus agalactiae, a Group B *Streptococcus* (GBS), produces a range of pathological conditions in aquatic animal species [1]. It causes disease in fish and humans [2] and animals, such as dogs, cats, and cattle [3]. GBS is the main causative agent of Streptococcosis outbreaks in freshwater fish aquaculture[4]. S. agalactiae has been associated with cases of Streptococcosis outbreaks among humans following fish consumption, indicating its potential as a zoonotic agent [5]. S. agalactiae is responsible for meningoencephalitis and septicemia in freshwater fish such as Tilapia [6], and cases of sudden death without detectable symptoms in freshwater, estuarine, and marine fish species [7]. Diseased fish show typical symptoms, including spiral swimming, anorexia, corneal opacity, unilateral or bilateral exophthalmia, and ulcers and hemorrhages in the skin [8,9]. Affected organs, such as the eye, liver, kidney, and brain, show gross pathological changes, enlarging and showing hemorrhagic and inflammation signs [10]. However, fish may show no signs before sudden death [11].

The severity of the disease produced may be attributable to factors such as its virulence marker and water temperature [12,13]. Virulence genes facilitate the ability of the pathogen to cause disease. They are classified into three classes: adhesin genes (fbsA, fbsB, pavA, lmb,and scpB), invasin genes (cylE, cfb, Spb1, hylB, rib, and bca), and immune evasion genes (bac, cspA,and pbp1A/ponA). They play a major role in pathogenicity after infection of the host [14]. These genes also help bacteria stay alive, make the host less angry, and let the bacteria get around the immune system.

The variation in susceptibility of S. agalactiae to antimicrobials could be due to variations in serotypes or to the repeated uncontrolled application of antibiotics in aquaculture [15]. Several antibiotics are used to eliminate streptococcal infection, but these antibiotics’ frequent and improper application can produce antimicrobial drug resistance [13,16]. The difference in the effects of antimicrobial treatment and vaccination may be attributed to the ability of streptococci to colonize and survive inside phagocytic cells, preventing their exposure to macrophages [17].

The Nile Tilapia (*Oreochromis niloticus*) is the world’s fourth most cultured fish species [18]. It is the main species cultured in Egypt, representing about 65.15% of Egyptian fish production [19], because of its rapid growth rate, good feed conversion, ability to survive in poor environmental conditions, resistance to disease, ease of spawning, palatability, and good consumer acceptance [20]. Large-scale production of farmed Tilapia increases the fish’s stress and, consequently, their susceptibility to disease [21]. Streptococcal outbreaks can cause major economic losses and are a major challenge for the development of the tilapia industry worldwide [22]. There is a little consideration about the resistant phenotypes, antimicrobial susceptibility, and virulence mechanisms of S. agalactiae recovered from farmed, diseased, and retailed Tilapia fish in Egypt.Thus, this study aimed to detect the prevalence of S. agalactiae among Tilapia fish recovered from diseased farmed fish and retail fish, study their antimicrobial susceptibility, and genotype their virulence-associated genes. Finally, to evaluate the hygiene measures taken among retail shop Tilapia fish. 

## Materials and Methods

### Ethical approval

Our study design and fish sampling were approved by the Research Ethics Committee of the Faculty of Veterinary Medicine, Mansoura University, Egypt (Protocol code: M/19).

### Fish sampling

From April 2020 to November 2020, 300 Nile Tilapia fish were collected. One hundred fish were sampled from farms located at EL-Manzala Lake, and two hundred fish were collected from retail markets in Dakahlia and Damietta provenances, Egypt. Fish samples were gathered in sterile screw-capped plastic vials, placed in an icebox, and sent for microbiological analysis at the Bacteriology, Mycology, and Immunology Department, Faculty of Veterinary Medicine, Mansoura University.

### Isolation of S. agalactiae from Tilapia

Under sterile conditions, swabs were sampled from the kidney, brain, liver, eye, spleen, and gills and plated on Edward agar (Oxoid, Basingstoke, UK) and 5% sheep blood agar (Oxoid, Basingstoke, UK), followed by incubation for 24 h at 37°C. Tryptone soy agar (Oxoid, Basingstoke, UK) and blood agar (Oxoid, Basingstoke, UK) were streaked with separate cultured colonies showing morphology similar to Streptococci and then incubated at 37°C for 24 h to obtain pure isolates. Streptococcal isolates were characterized using colonial morphology, Gram staining, motility, catalase tests, cytochrome oxidase tests, hemolysis on 5% sheep blood agar, and esculin hydrolysis on bile esculin slants [23,24]. The CAMP reaction was used as a presumptive diagnosis for S. agalactiae, as the CAMP factor was identified first in S. agalactiae [25]. All suspected streptococci isolates were preserved in 20% glycerol.

### DNA extraction

Genomic DNA was extracted from all strains via homogenizing of a few bacterial colonies (3–5 colonies) in about two hundred milliliters of deionized water, then boiling for fifteen minutes, then centrifugation at 10,000 *g* for three minutes. All the supernatant was collected in a sterile Eppendorf tube and used as a DNA template [26]. The genomic DNA was stored at −20°C until it was used.

### Molecular identification of S. agalactiae using PCR

All suspected Streptococcus spp. were characterized using polymerase chain reaction (PCR) analysis of specific primers targeting the S. agalactiae-specific dltS gene. The primer sequence and PCR cyclic conditions were mentioned by Poyart et al. [27] (Table 1). The PCR used reaction mix contained 12.5 µl 2× Tag PCR Master Mix (Enzynomics, Daejeon, South Korea), 1 µl of each primer (Metabion International AG, Steinkirchen, Germany), and 3 µl of template DNA in a final volume of 25 µl. The PCR cycle conditions were: 5 min at 94°C, 35 cycles at 94°C for 45 sec, 66°C for 45 sec, and 72°C for 90 sec, and the extension step at 72°C for 5 min using a thermal cycler (Applied Biosystem, 2720 thermal cycler, USA). Then, 10 µl of amplified PCR products were seen using 1% agarose gel through a gel documentation system (Hoefer PS300B life science, USA) and then checked with UV-induced fluorescence (Cleaver Scientific Ltd. UV gel documentation system, USA).

### Sequencing of the 16S rRNA of S. agalactiae

The primer sequence and PCR cyclic conditions for 16S rRNA used for S. agalactiae confirmation and sequencing were performed according to Lagacé et al. [28]. The purification of amplified products for strains was performed using QIAquick PCR Product Extraction Kits (Qiagen Inc. Valencia, CA)and sequenced using BigDye Terminator V3.1 cycle sequencing kits (Perkin-Elmer, Foster City, CA). According to the manufacturer’s instructions, the purification of the sequence reactions (Centrisep, spin-column) used an Applied Biosystems 3130 automated DNA Sequencer (ABI, 3130). BLAST^®^ analysis (Basic Local Alignment Search Tool)was used to identify the sequences and obtain their GenBank accession numbers [29]. Phylogenetic analyses were performed using maximum likelihood, neighbor-joining, and maximum parsimony in MEGA6 (Fig. 3) [30].

**Table 1.** Oligonucleotides and PCR cyclic conditions for PCR amplification of genes used in this study.

Target gene	Primer	Nucleotide sequence (5’-3’)	Target gene (bp)	PCR cyclic conditions	Reference
*Dlts*	*dlts-F*	AAGTACATGCTGATCAAGT	952	5 min at 94°C, 35 cycles at 94°C for 45 sec, 66°C for 45 sec, 72°C for 1.5 min, and 72°C for 5 min	[27]
	*dlts-R*	TCTTGATCAACTTGTTGTAC			
*16S rRNA*	*F27*	AGAGTTTGATCMTGGCTCAG	1485	5 min at 95°C, 35 cycles at 94°C for 30 sec, 56°C for 60 sec, 72°C for 60 sec, and 72°C for 10 min	[28]
	*R1492*	TACGGYTACCTTGTTACGACTT			
*Pbp1A/ponA*	*Pbp1A/ponA-F*	AGGGGTAGTAGCATTACCAT	939	5 min at 95°C, 38 cycles at 95°C for 30 sec, 47°C for 30 sec, 72°C for 30 sec, and 72°C for 10 min	[34]
	*Pbp1A/ponA-R*	CAACTATATGACTGGGATCG			
*Cfb*	*cfb-F*	GGATTCAACTGAACTCCAAC	600		
	*cfb-R*	GACAACTCCACAAGTGGTAA			
*FbsA*	*fbsA-F*	AACCGCAGCGACTTGTTA	278		
	*fbsA-R*	AAACAAGAGCCAAGTAGGTC			

### Antimicrobial susceptibility test for S. agalactiae

Using disc diffusion methods, all S. agalactiae strains were tested for susceptibility against 10 antimicrobial agents belonging to seven antimicrobial classes. These antimicrobial agents (Oxoid, Basingstoke, UK) were ciprofloxacin (CIP; 5 µg), penicillin (P; 10 µg), sulfamethoxazole/trimethoprim (SXT; 25 µg), ceftriaxone (CRO; 30 µg), tetracycline (TE; 30 µg), cefotaxime (CTX; 30 µg), erythromycin (E; 15 µg), ampicillin (AMP; 10 µg), imipenem (IPM; 10 µg), and gentamicin (CN; 10 µg) according to CLSI [31]. Tests were performed on Mueller–Hinton agar (Oxoid, Basingstoke, UK) supplemented with 5% sheep blood and incubated aerobically overnight at 37°C. The results were interpreted according to the Clinical and Laboratory Standards Institute [31]. Strains that showed resistance to three or more antimicrobial agents belonging to three different antibiotic classes were considered multi-drug resistance (MDR) bacteria [32]. A multiple antibiotic resistance (MAR) index was calculated according to Krumperman [33].

### Genotyping of virulence-associated genes using multiplex PCR

*Streptococcus* agalactiae strains were genotyped for the standard virulence genes fbsA (fibrinogen-binding protein FbsA), cfb (CAMP factor), and pbp1A/ponA (penicillin-binding protein 1A), which were amplified as described by Kannika et al. [34] using multiplex PCR (Table 1). The reaction mixture contained 12.5 µl of PCR Master Mix, 1 µl of each primer, and 3 µl of template DNA, made up with nuclease-free water to a final reaction volume of 25 µl. The amplification conditions were as follows: an initial denaturation of 5 min at 95°C, followed by 38 cycles of 95°C for 30 sec, 47°C for 30 sec, and 72°C for 30 sec, then a final extension of 10 min at 72°C (Table 1). The amplifications were examined on a thermal cycler (Applied Biosystem, 2720 thermal cycler, USA). The amplified PCR products were visualized on a 1% agarose gel using a gel documentation system (Hoefer PS300B life science, USA) and then checked using UV-induced fluorescence (Cleaver Scientific LTD UV gel documentation system, USA).

## Results

### Prevalence of S. agalactiae isolated from Tilapia

**Figure 1.**
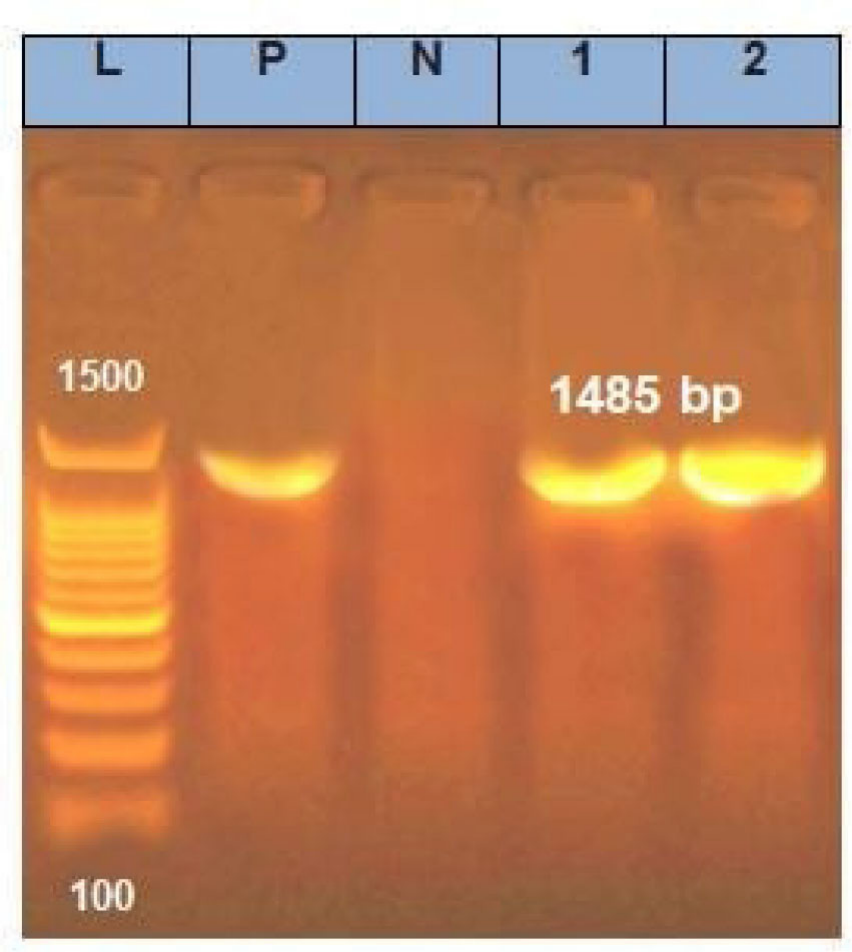
PCR analysis of *16SrRNA*. Lanes: 1—MW-DNA ladder (100bp); 2—positive control; 3—negative control; 4,5—*Streptococcus* agalactiae strains.

The Streptococcus species appeared as small, blueish colonies with a smooth, white edgeon Edward agar and translucent to slightly opaque, pinpoint colonies surrounded by a beta-hemolytic zone on blood agar. These bacteria were biochemically identified and shown to be Gram-positive cocci, non-motile, oxidase-positive, catalase-negative, and CAMP positive. Of the 300 cultured samples from freshwater fish (O. niloticus), 89 isolates (29.6%) were identified as Streptococcus spp. using routine phenotypic methods. The suspected Streptococcus isolates were subjected to molecular identification (PCR) using species-specific dlt-s targeting S. agalactiae; almost 23.6% (21/89) of the suspected isolates were confirmed as S. agalactiae. The overall prevalence rate of S. agalactiae in all examined Tilapia samples in this study was 7% (21/300). Streptococcus agalactiae was isolated from 13% (13/100) of diseased farmed Tilapia fish and 4% (8/200) of market Tilapia fish (Table 2, Fig. 1). The S. agalactiae strains were isolated from fish samples as follows: 10 (47.6%) from the liver; 6 (28.57%) from the kidney; 2 (9.5%) from each eye and gill samples; 1 (4.85) from spleen samples.

**Table 2.** Prevalence of *Streptococcus spp* isolated from market and farmed Tilapia fish samples using PCR assays.

Source	Farmed Tilapia Fish (No/%)	Market Tilapia Fish (No/%)	**Total Number (No/%**)
Overall number of Samples	100	200	300
Number of *Streptococcus* Isolates	34 (34%)	55 (27.5%)	89 (29.6%)
Number of *Streptococcus agalactia*	13 (13%)	8 (4%)	21 (7%)

### Antimicrobial susceptibility testing of S. agalactiae

Susceptibility tests to 10 antimicrobial agents from seven classes of antimicrobial agents showed that the most antimicrobial resistance of S. agalactiae strains was detected versus ampicillin at 95% (20/21) and erythromycin at 95%, followed by cefotaxime and trimethoprim-sulfamethoxazole (76% each), ceftriaxone (72%), tetracycline (66%), gentamicin (62%), and penicillin (57%). Less antimicrobial resistance was detected against ciprofloxacin, at 43% (9/21), and imipenem, at 5% (1/21) (Table 3). MDR has been observed in 90.5% of isolates tested. MDR was present in all S. agalactiae strains isolated from market Tilapia but in farmed Tilapia at lower levels, of 84.6%. Nineteen antibiotypes were identified in S. agalactiae. The most predominant antibiotypes through isolates were P, CN, SXT, CRO, TE, CTX, E, AMP, and CIP, P, CN, SXT, CRO, CTX, E, AMP, at 10.5% for each antibiotype (Table 4).

### Virulence associated genes in *S. agalactiae* strains

**Figure 2.**
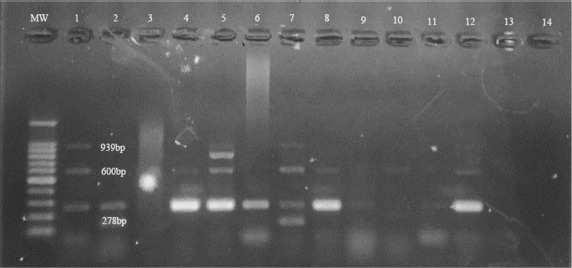
Multiplex PCR analysis of *Streptococcus agalactiae* virulence genes showed MW-DNA ladder (100 bp), *fbsA* gene (278 bp), *cfb* gene (600 bp), and *pbp1A/ponA* gene (939 pb).

Three virulence genes were used to predict the virulence of S. agalactiae: fbsA, cfb, and pbp1A/ponA. The fbsA gene was detected in all isolates, while cfb was present in 76% (16/21) of isolates. Around half the isolates (11/21; 52%) carried the pbp1A/ponA gene. The fbsA and cfb genes were recovered from sixteen isolates, and all 3 virulence genes were isolated from 11 isolates (Fig. 2; Table 5).

### Sequencing of the 16S rRNA of S. agalactiae

**Figure 3.**
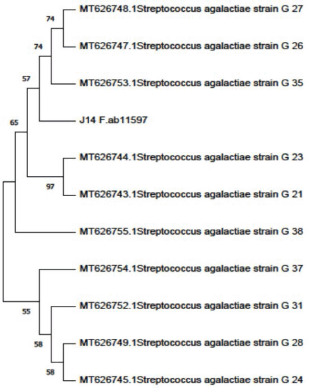
Phylogenetic trees viewing the genetic relatedness among *S. agalactiae* strains according to nucleotide sequence analysis of A) *16S-rRNA* gene (OL335944).

After identifying S. agalactiae strains for 16S rRNA using the PCR method, sequencing of S. agalactiae strains was applied by Elim biopharmaceuticals (USA). The nucleotide sequence of the S. agalactiae strains was deposited in GenBank under accession no. OL335944. Phylogenetic trees show the genetic relatedness among S. agalactiae strains according to nucleotide sequence analysis of the 16S-rRNA gene (Fig. 3). 

## Discussion

GBS is a common group of bacteria in humans and fish, causing meningitis in newborns, mastitis in cattle, and sepsis in rabbits [35]. *Streptococcus* agalactiae is the dominant species of streptococci associated with fish disease, especially in Tilapia production [36]. *Streptococcus* agalactiae causes high mortality in susceptible fish species, reaching 50%–70% in intensive farming systems [6]. Some serotypes of S. agalactiae from human newborns with meningitis and cattle result in disease and death in infected Nile Tilapia [6], causing severe public health problems. To the best of authors’ knowledge, there is a gap in knowledge considering the resistance mechanisms in S. agalactiae; studies from Egypt have not focused on the molecular characterization of GBS from O. niloticus to assess its environmental hazard. Our study clarifies the investigation of S. agalactiae in both diseased and retail Tilapia fish, antimicrobial resistance, and its role in transmitting virulence determinates between aquatic animals and humans via the food route.

**Table 3.** Antimicrobial susceptibility results of *Streptococcus agalactiae* strains isolated from fish (*n* = 21).

Antimicrobial Agent	Family	Disc code	Resistant No/(%)	Intermediate No/(%)	Susceptible No/(%)
Ampicillin	β-lactams	Amp	20/(95%)	Zero	1/(5%)
Penicillin		p	12/(57%)	Zero	9/(43%)
Imipenem		Imp	1/(5%)	1/ (5%)	19/(90%)
Erythromycin	Macrolide	E	20/(95%)	Zero	1/(5%)
Cefotaxime	Cephalosporin	CTX	16/(76%)	Zero	5/(24%)
Ceftriaxone		CRO	15/(72%)	Zero	6/(28%)
Trimethoprim/Sulphamethoxazole	Sulphonamide	SXT	16/(76%)	Zero	5/(24%)
Tetracycline	Tetracycline	TE	14/(66%)	5/(24%)	2/(10%)
Gentamicin	Aminoglycoside	CN	13/(62%)	Zero	8/(38%)
Ciprofloxacin	Fluoroquinoline	CIP	9/(43%)	9/(43%)	3/(14%)

**Table 4.** Antibiogram and MAR of *Streptococcus agalactiae* strains isolated from fish (*n* = 21).

	Resistance pattern	MAR index	Isolates No. (%)
I	CRO	0.1	1 (5.2)
II	P, E, AMP	o.3	1 (5.2)
III	P, CTX, E, AMP	o.4	1 (5.2)
IV	SXT, TE, CTX, E, AMP	0.5	1 (5.2)
V	P, SXT, TE, E, AMP	0.5	1 (5.2)
VI	P, TE, CTX, E, AMP	0.5	1 (5.2)
VII	CN, CRO, TE, CTX, E, AMP	0.6	1 (5.2)
VIII	CIP, CN, SXT, CRO, E, AMP	0.6	1 (5.2)
IX	CN, SXT, CRO, TE, CTX, E, AMP	0.7	1 (5.2)
X	CIP, SXT, CRO, TE, CTX, E, AMP	0.7	1 (5.2)
XI	CIP, P, CN, SXT, TE, E, AMP	0.7	1 (5.2)
XII	CIP, SXT, CRO, TE, CTX, E, AMP	0.7	1 (5.2)
XIII	CN, SXT, CRO, TE, CTX, E, AMP	0.7	1 (5.2)
XIV	CIP, CN, SXT, CRO, CTX, E, AMP	0.7	1 (5.2)
XV	P, CN, SXT, CRO, TE, CTX, E, AMP	0.8	2 (10.5)
XVI	CIP, P, CN, SXT, CRO, CTX, E, AMP	0.8	2 (10.5)
XVII	CIP, P, CN, SXT, CRO, TE, CTX, E, AMP	0.9	1 (5.2)
XVIII	P, CN, IPM, SXT, CRO, TE, CTX, E, AMP	0.9	1 (5.2)
XIX	CIP, P, CN, SXT, CRO, TE, CTX, E, AMP	0.9	1 (5.2)

The overall prevalence rate of S. agalactiae was 7% (21/300). The prevalence of diseased farmed Tilapia (13/100; 13%) was higher than that of market Tilapia (8/200; 4%). Previous results were consistent with ours in diseased farmed Tilapia [37]. However, a higher prevalence was found in studies of S. agalactiae from diseased fish from Saudi Arabia, Piura, Peru, and Thailand, of 44.4%, 56.25%, and 30.9%, respectively [34,38,39]. In China, S. agalactiae has been the causative agent for more than 90% of infected Tilapia since 2009 [40]. In Colombia and Brazil, S. agalactiae infection has high mortality in Tilapia farms [41,42]. Higher infection rates have been reported in Latin America and Asia [43]. In previous reports, fresh Nile tilapia showed a lower prevalence of infection than in our study [16,44]. In Egypt, a study from Kafr El-Sheikh, Egypt isolated S. agalactiae from Tilapia during the summer with a prevalence of 13% [45]. 

**Table 5.** Prevalence of virulence genes in *Streptococcus agalactiae* isolated from market and farmed Tilapia fish samples.

Virulence genes	Farmed Tilapia Fish No (%)	Market Tilapia Fish No (%)	Total number (%)
*fbsA* gene	13 (61.9%)	8 (38.09%)	21 (100%)
*cfb* gene	10 (47.62%)	6 (28.57%)	16 (76%)
*Pbp1A/ponA* gene	7 (33.33%)	4 (19.05%)	11 (52%)

The diversity of GBS prevalence can be attributed to multiple factors. Tilapia under unsuitable conditions is susceptible to several bacterial diseases, especially S. agalactiae. The contributing factors are high water temperature, high stocking density, and poor water quality due to high ammonia levels and low dissolved oxygen levels [6,46]. All outbreaks of streptococcosis in Nile Tilapia occurred in summer when the water temperature rose above 27°C [47]. High temperatures are a good environment for expressing the S. agalactiae virulence genes, leading to severe damage to fish tissue [47].

The rapidly growing antimicrobial resistance rate is a serious issue worldwide for human and veterinary medicine [43,48–51]. In our study, the susceptibility of S. agalactiae to10 antimicrobials from seven antimicrobial classes was tested. Of these antimicrobials, S. agalactiae isolates showed the most potent resistance against ampicillin (95%); however, lower resistance was detected in other reports [38,45]. The isolates studied exhibited greater resistance to tetracycline and penicillin (66% and 57%, respectively) than was reported in previous studies [34,38,39,45,52,53–55]. Similar tetracycline and penicillin resistance result was reported previously [16,22]. The drugs of choice for GBS treatment are penicillin and beta-lactams, followed by macrolides (erythromycin) used in individuals allergic to beta-lactams [56,57]. They are used in aquaculture for prophylactic or treatment purposes [58,59]. Almost all isolates were resistant to erythromycin, as reported by other authors [16,22,60]. The mechanism of resistance against erythromycin in the bacteria includes changes of the ribosomal target by a methylase [49,59]. It is important to search for other options for infection control [50,59].

In our study, a lower rate of sensitivity to trimethoprim/sulfamethoxazole (24%) was detected in our study. However, other studies found higher sensitivity rates [34,39]. Our results showed higher resistance against gentamicin (62%), as reported in several reports [15,21,51,59].However, other studies found lower levels of resistance [16,22]. Gentamicin affects the survival of bacteria by preventing the binding of its messenger RNA, which causes misreading of its DNA and changes to the proteins it makes.

Antimicrobial resistance against quinolones and third-generation cephalosporins was about 40% and 75%, respectively. This result was different from those of other studies into the antimicrobial sensitivity of S. agalactiae to third-generation cephalosporins [38,45]. Fluoroquinolone resistance has increased lately, and this increase has been attributed to many causes, such as efflux mechanisms or mutations in the quinolone-resistance-determining regions [54,55]. Non-guided and inaccurate use of antibiotics has resulted in many problems, such as resistance, and can affect the safety and quality of food [55,59]. This is a big problem for public health because of the spread of MDR in human communities, hospitals, animal farming, and veterinary medicine. It has become a problem for public health all over the world.

In this study, one of each of the three functional categories of virulence genes—adhesins, invasions, and immune evasions—was characterized in all isolates to determine their pathogenic and invasive abilities. At least two virulence genes were identified in the S. agalactiae isolates. The fbsA gene, responsible for an adhesion protein on the bacterial surface, promoted adhesion to and invasion of host cells [57,59] and was detected in our isolates. This gene was not expressed in all isolates in a study from Thailand [57,59]. The cfb gene, which encodes CAMP factor, a secreted pore-forming protein causing lysis of red blood cells, was carried by 76% of isolates. Thus, the hemolysis caused by the CAMP factor can be used for the phenotypic characterization of S. agalactiae. The penicillin-binding protein A encoding gene (pbp1A/ponA), which was detected in almost half of the isolates, leads to enhancement of the resistance of the host to antimicrobial peptides. Previous studies have seen the three virulence genes in all S. agalactiae isolates [34,44]. Understanding pathogenesis in aquatic outbreaks is complex because it is a process that involves both bacterial virulence genes and other related factors. Characterization of the patterns of virulence genes will help determine the genetic diversity of *Streptococcus* spp. and understand the genetic relatedness between bacterial virulence and host adaptations [60].

## Conclusions

Intensive cultivation systems with poor environmental conditions are related to the high mortality of fish, causing severe economic losses. Thus, special monitoring programs, efficient sanitary measures, and constant monitoring are essential in controlling and treating GBS. MDR is an emerging global threat, limiting our options for treatment of GBS infections due to uncontrolled use of antibiotics, which must be controlled to limit the emergence of MDR strains in the environment.

## List of abbreviations

GBS, Group B Streptococcus; MDR, Multi-drug resistance; DNA, Deoxyribonucleic acid; PCR, Polymerase Chain Reaction; CAMP, Christie Atkins Munch-Petersen; *fbsA*, fibrinogen-binding protein; *fbsB*, fibrinogen-binding protein; *pavA*, fibrinogen-binding protein; *scpB*, Segregation and condensation protein B; *Lmb*, laminin-binding protein; *cylE*, Β-haemolysin/cytolysin; Cfb, CAMP factor; *Spb1*, haemolysin III; *hylB*, Hyaluronatelyase; *Rib*, the surface protein; *Bca*, C-α protein; *Bac*, C-β protein; *cspA*, the serine protease; *Pbp1A/ponA*, penicillin-binding protein 1A.
